# *Physiological Reports* celebrates its first 10 years

**DOI:** 10.14814/phy2.15874

**Published:** 2023-11-28

**Authors:** Josephine C. Adams, Oliver J. Price, Natasha Rogers, Sharon Rounds, Akiyuki Taruno, Arianne L. Theiss, Shizuka Uchida, David C. Wright

**Affiliations:** ^1^ School of Biochemistry University of Bristol Bristol UK; ^2^ School of Biomedical Sciences, Faculty of Biological Sciences University of Leeds West Yorkshire UK; ^3^ Centre for Transplant and Renal Research Westmead Institute for Medical Research Westmead New South Wales Australia; ^4^ Department of Medicine, The Warren Alpert Medical School, VA Providence Health Care System Brown University Providence Rhode Island USA; ^5^ Department of Molecular Cell Physiology Kyoto Prefectural University of Medicine Kyoto Japan; ^6^ Division of Gastroenterology & Hepatology University of Colorado Anschutz Medical Campus Aurora Colorado USA; ^7^ Center for RNA Medicine, Department of Clinical Medicine Aalborg University Copenhagen SV Denmark; ^8^ School of Kinesiology University of British Columbia Vancouver British Columbia Canada; ^9^ Faculty of Land and Food Systems University of British Columbia Vancouver British Columbia Canada; ^10^ BC Children's Hospital Research Institute Vancouver British Columbia Canada

“Physiological Reports” was launched as a new Gold Open Access journal in 2013. The editors are delighted to celebrate 10 years of its publication through an upcoming “10th Anniversary Year.” In our capacity as the current team of Executive Editors since January 2023, we acknowledge and thank the Founding Editor‐in‐Chief, Sue Wray, and first Deputy Editor and second Editor‐in‐Chief, Thomas Kleyman, the co‐owning Societies American Physiological Society (APS) and The Physiological Society (TPS), the publisher Wiley, and other prior Editors and Editorial Board members for their collective inspiration and energy in starting Physiological Reports and growing its scope and reputation over the last decade. Separate Anniversary Editorials by Sue Wray and Tom Kleyman will provide their personal perspectives and insights into the early years. In following months, Editorials will also be contributed by several Editors representative of the APS and TPS transferring journals.

Physiological Reports was established as the first joint, and the first Open Access, journal of APS and TPS, with a mission to publish sound science in basic and translational physiology. Since the beginning, the journal has handled manuscripts transferred from APS, TPS and Wiley supporter journals as well as manuscripts received by conventional direct submission. As a fully Gold Open Access journal, over the last 10 years Physiological Reports has developed global outreach in the communication of physiological research. Downloads of full‐text articles have increased each year, and data from 2022 show download activity for full‐text articles published in Physiological Reports in most countries around the world. Between January 2021 and October 2022, published papers originated from 45 countries and, in 2023 to date, publications have originated from over 40 countries. The sound science mission of Physiological Reports has also proved compatible with excellent quality and robust citation: Individual papers achieve high citation, and the journal has achieved a new 2022 2‐year Journal Impact Factor of 2.5 and a CiteScore of 4.

It is apparent that authors have appreciated Physiological Reports as a journal of broad scope. The diversity of disciplines of publications is exemplified by the top‐cited articles published in each year (Table 1), and we extend our congratulations to the authors of these publications. Certain common themes are also evident, as highlighted in Figure 1, which is based on the titles of these papers. Overall, research into human physiology has emerged as a prominent topic of the journal, yet the research papers also report on a diversity of animal species. In addition to rodent models, the journal has published studies on agricultural animals, brown bears, camels, gray seal, birds, fish species, and *Drosophila*, among others. In vitro physiological models have expanded beyond conventional cell lines and primary cell strains to include stem cells, induced‐pluripotent stem cells, organoids and other “mini‐organ” forms of cell culture.

**TABLE 1:** The top two most‐cited original research articles, by year, published in Physiological Reports. Citations were analyzed by each year of publication.

Year of publication	Article title	Authors/doi
2013 2013	The truncated splice variants, NT‐PGC‐1α and PGC‐1α4, increase with both endurance and resistance exercise in human skeletal muscle Subcutaneous adipose tissue transplantation in diet‐induced obese mice attenuates metabolic dysregulation while removal exacerbates it	Ydfors et al., https://doi.org/10.1002/phy2.140 Forster et al., https://doi.org/10.1002/phy2.15
2014 2014	Salmonella‐infected crypt‐derived intestinal organoid culture system for host–bacterial interactions Nox‐4 deletion reduces oxidative stress and injury by PKC‐α‐associated mechanisms in diabetic nephropathy	Zhang et al., https://doi.org/10.14814/phy2.12147 Thallas‐Bonke et al., https://doi.org/10.14814/phy2.12192
2015 2015	Leaky intestine and impaired microbiome in an amyotrophic lateral sclerosis mouse model Strengthening of the intestinal epithelial tight junction by Bifidobacterium bifidum	Wu et al., https://doi.org/10.14814/phy2.12356 Hsieh et al., https://doi.org/10.14814/phy2.12327
2016 2016	Mitochondrial dysfunction and oxidative stress in patients with chronic kidney disease. The response of muscle protein synthesis following whole‐body resistance exercise is greater following 40 g than 20 g of ingested whey protein	Gamboa et al., https://doi.org/10.14814/phy2.12780 Macnaughton et al., https://doi.org/10.14814/phy2.12893
2017 2017	Gut inflammation and dysbiosis in human motor neuron disease. Chronic intermittent hypoxia induces oxidative stress and inflammation in brain regions associated with early‐stage neurodegeneration.	Rowin et al., https://doi.org/10.14814/phy2.13443 Snyder et al., https://doi.org/10.14814/phy2.13258
2018 2018	Empagliflozin normalizes the size and number of mitochondria and prevents reduction in mitochondrial size after myocardial infarction in diabetic hearts Effects of short‐term endurance exercise on gut microbiota in elderly men	Mizuno et al., https://doi.org/10.14814/phy2.13741 Taniguchi et al https://doi.org/10.14814/phy2.13935
2019 2019	The maximal metabolic steady state: redefining the “gold standard.” Dynamic cerebral autoregulation is attenuated in young fit women	Jones et al., https://doi.org/10.14814/phy2.14098 Labrecque et al. https://doi.org/10.14814/phy2.13984
2020 2020	Osmotic diuresis by SGLT2 inhibition stimulates vasopressin‐induced water reabsorption to maintain body fluid volume Mechanisms of muscle insulin resistance and the cross‐talk with liver and adipose tissue	Masuda et al., https://doi.org/10.14814/phy2.14360 Silva Rosa et al., https://doi.org/10.14814/phy2.14607
2021 2021	SARS CoV‐2‐related microvascular damage and symptoms during and after COVID‐19: Consequences of capillary transit‐time changes, tissue hypoxia, and inflammation Losing the dogmatic view of cerebral autoregulation	Østergaard, https://doi.org/10.14814/phy2.14726 Brassard et al., https://doi.org/10.14814/phy2.14982
2022 2022	The role of astrocytes in epileptic disorders Classification and occurrence of an abnormal breathing pattern during cardiopulmonary exercise testing in subjects with persistent symptoms following COVID‐19 disease	Hayatdavoudi et al., https://doi.org/10.14814/phy2.15239 Gruenewaldt et al., https://doi.org/10.14814/phy2.15197

**FIGURE 1:**
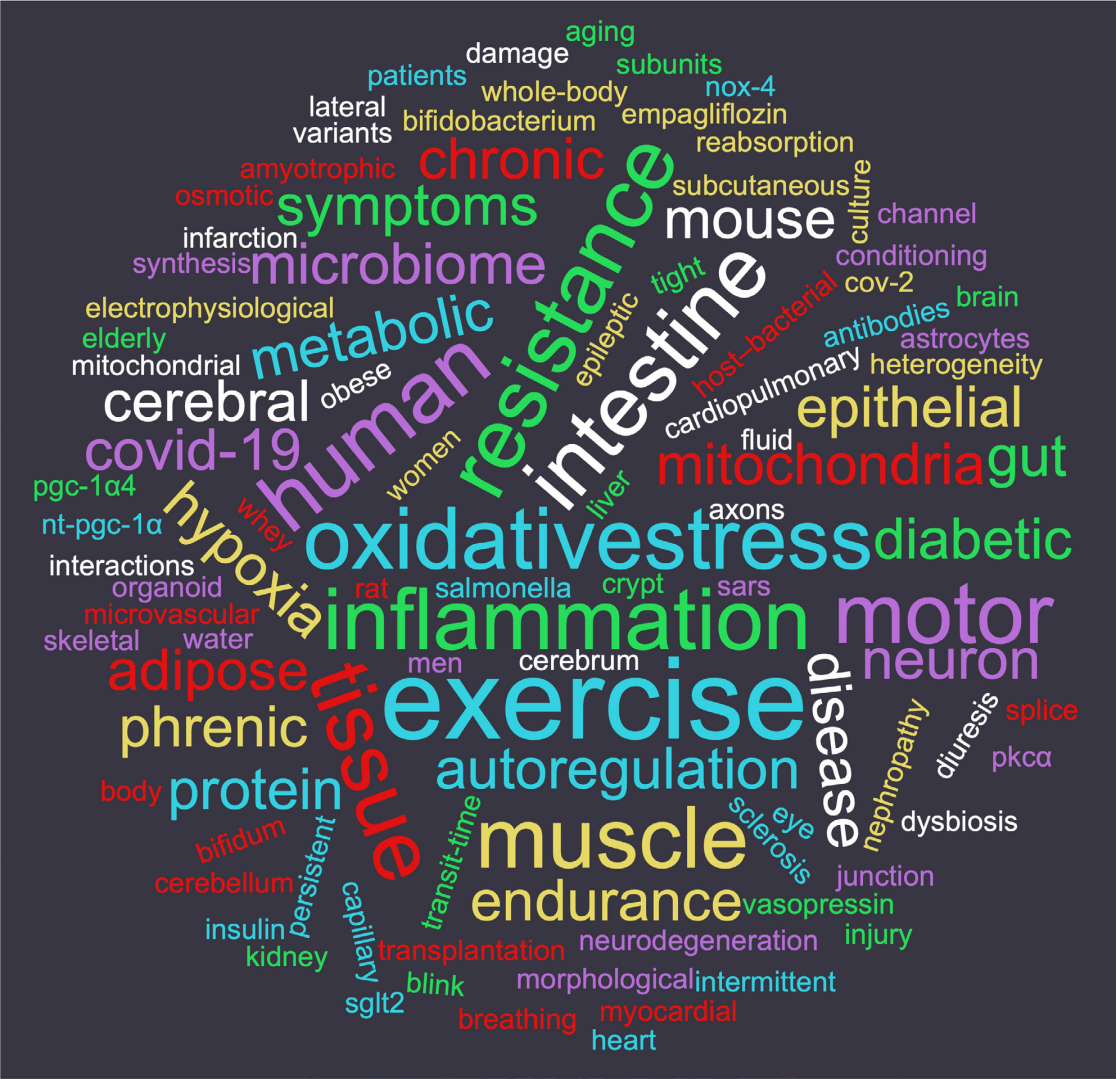
Prominent research topics of top‐cited Physiological Reports publications. The wordcloud is based on the titles of the two most‐cited papers published in each year, as shown in Table 1. Generated online at https://www.wordclouds.co.uk/.

The 10th Anniversary Year will run from December 1, 2023, to November 30, 2024, during which new anniversary logos will provide extra visibility to this landmark time, to associated journal activities, and to articles published during this year. In addition to the special Editorials mentioned above, we will celebrate the first decade of Physiological Reports with new or enhanced publication activities. Over the first 10 years, the focus has been on publishing original research articles, case reports, and occasional review articles. We have now set up Methods articles as a distinct category and look forward to future high quality submissions in this category (Adams, 2023 and see article details at https://physoc.onlinelibrary.wiley.com/hub/journal/2051817x/about/author‐guidelines/methods_in_physiology). We will also feature an expanded schedule of targeted Calls for Papers, starting with a Call for Papers related to the theme of “Exercise and Diet” (already open). Information about all Calls for Papers can be found at the journal website (https://physoc.onlinelibrary.wiley.com/hub/journal/2051817x/call‐for‐papers).

Physiological Reports supports the physiological research community, especially early‐career researchers, through poster or short talk awards made at major conferences (for recent awards see https://physoc.onlinelibrary.wiley.com/hub/journal/2051817x/awards). To celebrate the first 10 years, we are inaugurating a “Paper of the Year” award that will recognize the research papers of highest sound science quality published at Physiological Reports. Original research papers with single first authors will be ranked by the executive editors in a two‐step process. The first award will be based on research papers published in the year December 2022 to November 2023, with the winner to be announced early in 2024.

The success of Physiological Reports over its first 10 years prompts the question “What will the next decade bring?” As we progress through the 10th Anniversary Year, we will look ahead to the future of Physiological Reports and the wider future of physiological research. With this in mind, and to foster engagement with the next generation of researchers, we are planning a “Short Review” writing competition for early‐career researchers (i.e., graduate research students or postdoctoral researchers within 5 years of the terminal degree), with the winning articles to be published in Physiological Reports. Further details of this exciting initiative will be disseminated in early 2024. We also plan to enhance the publication of invited Review articles, not only to emphasize major research areas of publications over the last 10 years and highlight current topics, but also to address emerging or expanding areas of physiology. For example, we are interested to feature the roles of new technologies in driving new research approaches, or pathophysiological conditions that present growing challenges, such as the increasing physiological stresses posed by the climate crisis and air pollution.

The vitality of a journal is driven not only by Editors' initiatives but also by the community it serves. We look forward to engaging with existing and new authors, readers and reviewers through these plans, and to ensuring the continued success and development of Physiological Reports into its second decade and beyond. Information about the aforementioned initiatives will be presented at the journal's website and through social media communications.

## FUNDING INFORMATION

No funding was associated with the preparation of this Editorial.

## CONFLICT OF INTEREST STATEMENT

There are no conflicts of interest to declare.

## ETHICS STATEMENT

The authors form the Executive Editorial team of Physiological Reports and were blinded from reviewing or making decisions for the manuscript. An alternate editor oversaw the manuscript process for this article.

## Data Availability

The data can be found within this article.
